# Evaluation of dietary curcumin nanospheres as phytobiotics on growth performance, serum biochemistry, nutritional composition, meat quality, gastrointestinal health, and fecal condition of finishing pigs

**DOI:** 10.3389/fvets.2023.1127309

**Published:** 2023-03-08

**Authors:** Mohammad Moniruzzaman, Dahye Kim, Hyunsoo Kim, Nayoung Kim, Sungyeon Chin, Adhimoolam Karthikeyan, Kyuhyuk Han, Taesun Min

**Affiliations:** ^1^Department of Animal Biotechnology, Jeju International Animal Research Center, Sustainable Agriculture Research Institute (SARI), Jeju National University, Jeju, Republic of Korea; ^2^Division of Animal Genetics and Bioinformatics, National Institute of Animal Science (NIAS), Rural Development Administration (RDA), Wanju, Republic of Korea; ^3^Subtropical Horticulture Research Institute, Jeju National University, Jeju, Republic of Korea; ^4^AT. Consulting, Hanlim-eup, Jeju, Republic of Korea; ^5^Department of Animal Biotechnology, Bio-Resources Computing Research Center, Sustainable Agriculture Research Institute (SARI), Jeju National University, Jeju, Republic of Korea

**Keywords:** curcumin nanospheres, growth, serum biochemistry, immunohistochemistry, malodors, microbes, meat quality, finishing pigs

## Abstract

Curcumin is a bioactive functional feeding stimulant that is widely used as an additive in cuisine and animal feeds. Owing to its hydrophobic nature and low bioavailability, the nanoformulation of curcumin has recently received special attention from researchers. In this study, we investigated the effects of curcumin nanospheres (CN) on the growth performance, serum biochemistry, meat quality, intestinal immunohistochemistry, fecal malodors and microbes in finishing pigs. A total of 90 crossbred pigs (Duroc × [Yorkshire × Landrace]) with an average initial body weight of 73.77 ± 0.08 kg were randomized into 3 dietary groups in triplicate pens (10 pigs in each pen): control (CON) without supplementation of CN and the pigs in the remaining two groups were supplemented with CN at 1.0 (CN1) and 2.0 (CN2) mL/kg diet for a 40-day long experiment. The results showed that pigs fed the higher CN supplemented diet (CN2) had significantly higher final weight (FW) and weight gain (WG) than those fed the CON diet, and no significant differences were observed in the feed conversion ratio (FCR) and average daily feed intake (ADFI) after 28 days. At the end of the experiment, pigs fed the CN supplemented diet showed no significant difference in WG, ADFI or FCR compared to those on the CON diet. Overall, at the termination of the 40-day feeding trial, dietary CN had a significant effect on FW and WG, except for ADFI and FCR, in finishing pigs. After 40 days of the feeding trial, serum biochemical parameters such as glutamic-pyruvic transaminase, glutamic-oxaloacetic transaminase, triglycerides, and total cholesterol levels were significantly decreased in pigs fed the CN supplemented diet. However, high density lipoprotein levels were significantly increased in pigs fed the CN diets. Protein and lipid contents, as well as yellowness and lightness of the neck and longissimus dorsi muscles were not significantly affected by CN supplementation; however, there was a tendency to increase the redness of the longissimus dorsi muscle in pigs fed the CN2 supplemented diet compared to the CON diet. Meat grading and carcass weight significantly increased in pigs fed a higher CN supplemented diet. Fecal *Escherichia coli* and ammonia gas were significantly depleted in pigs fed CN diets. Histomorphological parameters, such as villus height, crypt depth and goblet cells in the jejunum of the intestine were significantly increased in pigs fed CN diet. Immunohistochemical staining showed that pro-inflammatory cytokine like tumor necrosis factor-α expression was reduced in pigs fed CN supplemented diets compared to the CON diet; however, antibodies such as immunoglobulin A and tight junction proteins such as claudin 3 were highly expressed in the intestine of pigs fed the CN diets. Overall, the results demonstrate the potential of dietary curcumin nanospheres as a nanobiotechnology tool as well as an effective feed additive for improving the performance and health status of finishing pigs.

## Introduction

Curcumin is a polyphenolic bioactive compound extracted from the turmeric plant, *Curcuma longa*, which has phytotherapeutic potential in terms of its antimicrobial, antioxidant, anti-inflammatory, and immunostimulatory properties. It can be used as a nutritional supplement or phytobiotic in the diet of animals because of its pharmacological properties, especially as a growth promoter, immunomodulator and gastro-protector (1, 2). The most important functional property of curcumin is its highly safe and non-toxic nature, making it sutable for biomedical applications (3). Interestingly, no studies have reported the toxic effects of curcumin in humans or animals at higher levels of supplementation (3). Despite the non-toxic and beneficial nature of curcumin, the drawbacks of native curcumin is its hydrophobic (insoluble in water) nature and its oral bioavailability in human or animals is very low. This is a major challenge for supplemention of curcumin in the diets as an additive or as a drug from a clinical perspective. It has been reported that bioaccumulation of native curcumin levels in blood and tissues were very poor in hepatic and intestinal metabolism, and dietary curcumin is rapidly eliminated from the body through excreta, which ultimately lowers the bioavailability of native curcumin in organisms (4, 5). In this regard, nanoformulation of native curcumin could be a better option for higher utilization of curcumin in humans or animals in terms of making native curcumin more soluble and bioavailable. Several dosage forms have been proposed for the nanoformulation of curcumin, such as surfactant micelles, microemulsions, nanoemulsions, emulsions, solid lipid nanoparticles (SLNs), nanostructured lipid carriers (NLCs), biopolymer nanoparticles, and microgels, which show higher water solubility, chemical strength, and bioavailability of curcumin (6). For instance, Tabatabaeain et al. (7) postulated that *Satureja khuzistanica* essential oil loaded SLNs modified with chitosan folate (as NLCs) can be useful to enhance the bioavailability and degradability as well as encapsulation efficiency of the hydrophobic essential oils. Shaikh et al. (3) reported that the bioavailability of nanoencapsulated curcumin on oral administration was 9-times higher compared to native curcumin. In addition, Jaguezeski et al. (8) found that the dietary incorporation of curcumin nanocapsules can enhance milk quality by increasing the antioxidant activity and reducing lipid peroxidation in milk, where curcumin nanocapsules were used at 10-fold lower doses than native curcumin in the diet of dairy sheep. Furthermore, Marchiori et al. (9) postulated that dietary nanoencapsulated curcumin (10 mg/kg of feed) can improve the egg quality of quail using 3 times lower concentration than that of normal curcumin (30 mg/kg of feed) under cold stress conditions.

Curcumin in nanoencapsulated form is effective for growth and immunity enhancement in poultry, livestock and aquatic animals (8–15). However, information on the application of nanocurcumin in animal health is scarce compared to that on human health (4). In our previous studies, we reported the physical and chemical characteristics of curcumin in nanoencapsulated form, hereafter, curcumin nanospheres (CN), as well as findings of *in vitro* and *in vivo* studies using cell lines and murine models, respectively (16, 17). Recently, we discovered the efficacy of dietary CN in weaned piglets, where we demonstrated that dietary supplementation with CN could improve growth performance, feed utilization, and immune functions, as well as deplete the fecal infectious bacteria and ammonia gas discharge in piglets (12). Based on our previous findings, in the present study, we hypothesized that dietary CN would have potential effects on the growth performance, serum biochemistry, meat composition and quality, pro-inflammatory cytokines, gut barrier junction protein, and antibody expression in the jejunum of the intestine, as well as fecal noxious gases and pathogenic bacterial contents in finishing pigs.

## Materials and methods

### Ethics statement

Experimental protocols describing the management and care of animals were reviewed and approved by the Animal Care and Use Committee of Jeju National University, Republic of Korea (approval no. 2021-0056). Samples from the animals were taken carefully to minimize suffering and slaughtered humanely at the end of the experiment.

### Chemicals and kits

Curcumin powder (99% purity) extracted from the turmeric plant, *Curcuma longa* Linn, and lecithin (L-α-phosphatidylcholine) were purchased from Sigma-Aldrich (St. Louis, MO, USA). Toluene and dichloromethane (DCM) as the organic solvents were collected from Sigma-Aldrich (St. Louis, MO, USA) and Acros Organics (Janssen Pharmaceuticals, Geel, Belgium), respectively. The rest of the kits used were: goat anti-porcine immunoglobulin A (IgA) secondary antibody (NB724, Novus Biological, Abingdon, UK), horse anti-goat immunoglobulin G (IgG) antibody (BA-9500, Vector Laboratories, Inc., Burlingame, CA, USA), and bovine serum albumin (Sigma-Aldrich, St. Louis, MO, USA), the rest were purchased from Abcam (Abcam, Waltham, CA, USA) such as hematoxylin and eosin staining kit (ab245880), 3,3'-diaminobenzidine (DAB) detection immunohistochemistry (IHC) kit (ab64261), goat anti-rabbit IgG (ab6721), recombinant anti-TNF-α (tumor necrosis factor- alpha) antibody (ab270264), claudin 3 (CD3) monoclonal antibody (ab135372), and normal goat serum (ab7481). All other reagents used in the present study were laboratory grade.

### Curcumin nanospheres

The production protocol for CN and its physico-chemical characteristics have been previously described by our research group (12, 16, 17). Briefly, 200 mg of commercial curcumin powder were mixed with 40 mL of toluene, and the mixture was poured into 2 L of distilled water. The mixture was then sonicated at 50 kHz for 4 h (Sonictopia, Rep. of Korea) with continuous stirring. After sonication, toluene was fully evaporated from the mixture using a rotary evaporator (Buchi AG, Meierseggstrasse, Flawil, Switzerland) at 40°C. The concentrated liquid without toluene was then freeze dried for 72 h to obtain curcumin powder in nanoform (hereafter, nanocurcumin). An aliquot of 40 mg of nanocurcumin was added to a mixture of 200 mg phosphatidylcholine (lecithin) and 40 mL dichloromethane. In this study, lecithin was used to coat the nanocurcumin in the mixture. The CN solution was produced continuously and maintained at −70°C until use in pig feeds.

### Animals and diets

A total of 90 crossbred pigs (Duroc × [Landrace × Yorkshire]) with an average initial body weight of 73.77 ± 0.08 kg (average age 112 days) were reared in a regional commercial pig farm (Bada Pig Farm, Hanlim, Jeju, Korea) for 40 days. The finishing pigs were arbitrarily distributed into nine pens based on average body weight using a randomized block design (RBD) according to three dietary treatments. Each treatment consisted of three replicate pens, each of which contained 10 pigs (30 pigs per treatment). In the present study, we used commercial pig feed for the supplementation of CN in the experimental diets of pigs (Neopigg, Purina Korea). Three diets were designed as control (CON) without supplementing CN, one diet was supplemented with 1 mL CN/kg diet (CN1), and the diet of the remaining group was supplemented with 2 mL CN/kg (CN2). The CN solution was mixed with the commercial mash feed using a stainless steel mixer. The amount of CN supplemented in the diets and the preparation of the diets were based on previous studies (10, 12). The pigs were reared in concrete-floored pens with steel fencing, including the entrance. Each pen was provided with a self-feeding and drinking water system in a controlled environment with proper air supply and temperature (28°C). Pigs were allowed continuous access to feed and drinking water. All the pigs were ear tagged with a number to identify them individually for recording the sampling data.

### Growth and feed utilization

Growth and feed utilization in finishing pigs were measured in two stages: (i) Days 1–28 and (ii) Days 29–40. Pig weights and feed consumption data were measured, growth and feed utilization were calculated to obtain the final weight (FW), weight gain (WG), average daily gain (ADG), average daily feed intake (ADFI) and feed conversion ratio (FCR) after each stage. The overall growth performance and feed utilization were calculated/assessed after the end of the 40-day experiment.

### Serum biochemistry

For serological analyses, an aliquot of 5 ml of whole blood sample was obtained from the jugular vein of each pig using a non-heparinized Vacutainer syringe (24 gauge) and stored in a tube (Becton Dickinson Vacutainer Systems, Franklin Lakes, NJ, USA). Whole blood samples were collected early in the morning at day 28 and day 40 randomly based on the visual average size of the randomly selected pigs (three pigs from each pen) according to the three treatment groups (nine pigs per treatment). The obtained whole blood samples were left at room temperature (RT) for 10 min and centrifuged at 1,500 × g at 4°C for 20 min. After centrifugation, blood cells were precipitated, and the transparent supernatant of the blood (serum) was obtained using a micropipette and stored in Eppendorf tubes at −20°C for further analyses. Serological parameters such as glutamic-pyruvic transaminase (GPT), glutamic-oxaloacetic transaminase (GOT), bilirubin (BIL), triglycerides (TG), alkaline phosphatase (ALP), high-density lipoprotein (HDLP), cholesterol (CHOL), and blood urea were determined using Refloton kits compatible with a serolocial diagnostic machine (Refloton Plus, Hoffmann-La Roche, Rotkreuz, Switzerland).

### Meat quality, nutritional composition, and carcass grading

At the termination of the 40-day experiment, finishing and marketable pigs were transported from the commercial pig farm to a regional government approved pig slaughterhouse (Nonghyup Slaughterhouse, Hanlim, Jeju, Korea). Pigs were slaughtered by the stunning process and bleeding by cutting the head, and visceral parts were collected by evisceration. Intestinal parts (three samples from three pigs per pen of the treatments) were separated for further microbial and histological studies. Carcass weight and backfat thickness of the pigs were determined according to Kim et al. (18). A part of the neck and longissimus dorsi muscle at the 10th rib (~250 g each) (three samples from three pigs for each pen of the treatments) was collected, and meat color reading was performed at least at three sites of the respective sample based on lightness (L^*^), redness (a^*^), and yellowness (b^*^) by placing the measuring head vertically above the muscle samples using a digital chroma meter (8 mm aperture size, diffuse illumination/0< ° viewing angle, and illuminant type C) using the Minolta CR-410 instrument (Minolta CR-410, Konica Minolta Sensing Inc., Osaka, Japan). The neck and longissimus dorsi muscle samples were stored at −20°C for proximate composition analysis. The carcass quality of pork was graded as “Grade 1+,” “Grade 1,” or “Grade 2,” based on marbling, lean color, and conditions of belly streaks of pork (19).

The proximate composition of pork meat in terms of protein, lipid, ash and moisture contents of neck and longissimus muscle samples based on the dietary treatments was analyzed using the conventional methods followed by AOAC (20). Briefly, representative test samples (1 g each) were dried at 135°C for 3 h in a dryer to measure the moisture content of the meat samples. The ash content of the meat samples was obtained by burning the samples at 550°C in a muffle furnace. The Kjeldahl method was used to obtain the nitrogen (N) content in the meat samples (0.1 g), and the crude protein was measured through acid digestion, distillation, and titration of the samples using the formula, N × 6.25. The crude lipid content of the neck and longissimus dorsi meat samples (1 g each) was obtained by the ethyl-ether extraction method using a lipid extraction unit (Soxhlet apparatus 1,046, Tacator AB, Hoganas, Sweden).

### Intestinal and fecal microbial contents

From the collected intestinal parts of slaughtered pigs, approximately 5 cm sections were cut from the middle of the small intestine (jejunum), separated and stored in a plastic falcon tube under chilling conditions in an ice box for microbial analysis in the laboratory. For fecal microbial content, fresh fecal samples were obtained from every pen and collected in plastic zipper bags marked with diet numbers, and all zipper bags were stored in an ice box. Microbial analyses of the jejunum and feces were performed immediately after collection from the site. For this, 1 g of feces and 1 g of intestinal pieces (3 pigs from each pen; total 9 pigs for each treatment) were mixed with 9 ml of peptone water (CM0009; Oxoid Ltd, Basingstoke, Hampshire, UK), and homogenized by vortexing, and the supernatant of was collected by pipetting. The decimally (10 fold) diluted fecal and intestinal sample solutions were then poured onto MacConkey (CM0115, No.3; Oxoid Ltd, Basingstoke, Hampshire, UK) serial agar plates and incubated at 37°C for 24 h. The viable number of pathogenic bacteria, *Escherichia coli* in the fecal and intestinal samples were counted and calculated the number of bacterial colonies using a colony counter (Suntex automatic colony counter, Taiwan). Likewise, the *Lactobacillus* spp. in fecal and intestinal samples were identified on de Man-Rogosa-Sharpe (MRS) agar (CM0361; Oxoid Ltd., Basingstoke, Hampshire, UK) after incubation at 37°C for 48 h. Furthermore, the *Salmonella* spp. in fecal and intestinal samples were identified on Salmonella-Shigella (SS) agar (CM0099; Oxoid Ltd., Basingstoke, Hampshire, UK) after incubation at 37°C for 24 h.

### Fecal gas contents

Fecal gas analysis was performed based on the method described by Moniruzzaman et al. (12), with slight modifications. Briefly, the fecal samples (250 g each) in plastic zipper bags were kept in airtight conditions at RT (25°C) for 24 h. Then, the bags were connected using a hose pipe to the probes of specific gas sampling kits for the measurement of noxious gases such as ammonia (NH_3_) and hydrogen sulfide (H_2_S) in the feces of finishing pigs. The NH_3_ and H_2_S gases in the feces of pigs were measured in parts per million (ppm) using colorimetric analysis kits with a portable Gastec instrument (model GV-100S; Gastec Corp., Tokyo, Japan).

### Intestinal histomorphology

Histomorphological studies of the jejunum of finishing pigs fed with or without curcumin nanospheres were conducted using a standard protocol (21). Briefly, small sections (5 cm) of the middle parts of the small intestine (jejunum) from the eviscerated pigs of each treatment group (three pigs per pen) were cleaned with water and fixed in 4% paraformaldehyde for 24 h at 4°C. Gut samples were further cleaned, infiltrated, and dehydrated with different alcohols at different concentrations. The tissue sections along with the paraffin cubes were sliced (4 μm thickness) using a sharp cutter adjusted with a histological microtome machine (HistoCore, Leica Biosystems, Buffalo Grove, IL, USA), and hematoxylin and eosin (H&E) was used for staining (H&E, ab245880, Abcam, Cambridge, UK) of the tissue sections. The tissue sections were mounted with Canada balsam (mountant) and closely monitored using an Olympus light microscope (AX70 Olympus, Tokyo, Japan) fitted with a digital camera (DIXI Optics, Daejeon, Republic of Korea) to capture the tissue images. These images were then examined using the Image J operating system (Image J 1.32j, National Institute of Health, Bathesda, MD, USA). To measure the measurement of the villus height, crypt depth, villus height/crypt depth, intestinal muscular thickness and goblet cell number per villus, at least six images were obtained from each slide in pooled form with triplicate slides for each treatment.

### Intestinal immunohistochemistry

Tissue sections from the jejunum were deparaffinized with xylene and graded alcohols. The tissue sections were then treated with 3% H_2_O_2_ in methanol and 0.3% Triton X-100 was added for tissue permeabilization for 30 min. Tissue sections were further blocked in 20% normal goat serum (ab7481, Abcam, Cambridge, UK), and antigen retrieval was performed using trypsin antigen retrieval solution (ab970, Abcam, Cambridge, UK) at 37°C for 30 min. The tissue sections were incubated with antibodies, such as goat polyclonal anti-porcine IgA (NB724, Novus Biologicals, Centennial, CO, USA), recombinant anti-TNF alpha antibody (ab270264, Abcam, Cambridge, UK), and claudin 3 (CD3) monoclonal antibody (ab135372, Abcam, Cambridge, UK) overnight at 4°C according to the manufacturer's protocol. The tissue sections were washed with PBS buffer four times and incubated with secondary antibody, biotinylated goat anti rabbit IgG (H+L) (ab6721, Abcam, Cambridge, UK) for 10 min at RT. The sections were washed four times in PBS, and streptavidin peroxidase was applied and incubated for 10 min at RT. Finally, 1 ml of 3,3'-diaminobenzidine (DAB) substrate was added to 20 μl DAB chromogen (ab64261, Abcam, Cambridge, UK), mixed by swirling, and applied to the tissue sections. The tissue sections were further incubated for 10 min and counterstained with hematoxylin. The expression of IgA, TNF-α, and CD3 was quantified using the Image J program (Image J 1.32j, National Institute of Health, Bathesda, MD, USA). To obtain data on the expression of TNF-α, IgA, and CD3 in the jejunum of finishing pigs, at least six images from each histological slide were pooled for statistical analysis in triplicate.

### Statistical analysis

For statistical analysis, the pig pen mean values were expressed in triplicates. Normality and homogeneity of variance were assessed for arcsine-transformed percentage data using the Shapiro–Wilk and O'Brien tests, respectively. The data were initially analyzed using two-way analysis of variance (ANOVA) to check the interaction effect between the treatments and replication pens. As no significant interaction effects were found among the treatments and replication pens, we conducted a one-way ANOVA to check the dietary effects of CN supplemented or control diets in finishing pigs. Tukey's honestly significant difference (HSD) *post-hoc* test was used to check the significance of the treatment means. The effects of treatment means were analyzed based on a significance level of *P* < 0.05. All statistical analyses were performed using SAS version 9.1 operating system (SAS Institute, Cary, NC, USA).

## Results

### Dietary effects of CN on performance and feed usage in finishing pigs

The effects of CN on the growth performance and feed utilization of finishing pigs are shown in Table 1. The growth performance data showed that pigs fed the CN2 diet had significantly greater FW, WG and ADG than those fed the CON diet at the end of the 28-day rearing period. However, there were no significant differences in FW, WG, and ADG of pigs fed the CON and CN1 diets or the CN1 and CN2 diet groups. At the end of remaining 10 days (from 29 to 40 days), the results showed that dietary CN had no significant effects on WG, ADG, ADFI, and FCR in finishing pigs. Collectively, at the end of the 40 days of feeding trial in finishing pigs, we found significant effects of dietary CN on FW, WG, and ADG in pigs; however, no significant effect was observed in ADFI and FCR of pigs fed CN supplemented diets compared to the CON diet.

**Table 1:** Dietary curcumin nanospheres (CN) on performance and feed utilization in finishing pigs for 40 days^1^.

**Items**	**Dietary treatments**			***P*-value**
	**CON (no CN)**	**CN1 (1.0 ml/kg)**	**CN2 (2.0 ml/kg)**	
**Days 1–28**				
FW (kg)^2^	92.6 ± 0.2^b^	94.3 ± 0.7^a, b^	95.5 ± 1.6^a^	0.0345
WG (kg)^3^	18.8 ± 0.2^b^	20.5 ± 0.7^a, b^	21.7 ± 0.7^a^	0.0345
ADG (kg/d)^4^	0.67 ± 0.01^b^	0.73 ± 0.03^a, b^	0.78 ± 0.06^a^	0.0354
ADFI (kg/d/pig)^5^	1.9 ± 0.4^a^	1.9 ± 0.2^a^	1.7 ± 0.2^a^	0.5414
FCR^6^	2.8 ± 0.7^a^	2.6 ± 0.3^a^	2.1 ± 0.2^a^	0.2268
**Days 29–40**				
WG (kg/pig)	10.0 ± 0.9^a^	14.8 ± 3.5^a^	15.1 ± 1.4^a^	0.0536
ADG (kg/pig)	0.83 ± 0.1^a^	1.24 ± 0.3^a^	1.26 ± 0.1^a^	0.0554
ADFI (kg/d/pig)	2.0 ± 0.3^a^	2.2 ± 0.1^a^	2.1 ± 0.3^a^	0.5808
FCR	2.4 ± 0.4^a^	1.8± 0.4^a^	1.7± 0.2^a^	0.0886
**Days 1–40**				
FW (kg/pig)	102.6 ± 0.9^b^	109.1 ± 3.5^a^	110.6 ± 1.4^a^	0.0101
WG (kg/pig)	29.2 ± 0.2^b^	34.5 ± 4.7^a^	36.9 ± 1.9^a^	0.0106
ADG (kg/pig)	0.72 ± 0.02^b^	0.88 ± 0.09^a^	0.92 ± 0.03^a^	0.0106
ADFI (kg/d/pig)	2.7 ± 0.6^a^	2.8 ± 0.3^a^	2.5 ± 0.4^a^	0.7237
FCR	2.7 ± 0.6^a^	2.2 ± 0.1^a^	1.9 ± 0.2^a^	0.1146

^1^Values are mean ± SD from three replicate groups of pigs (*n* = 3), where mean ± SD in each row with different superscripts (a,b) are statistically significant at the 5% level of significance (*P* < 0.05).

^2^Final weight; ^3^Weight gain = (final weight -initial weight)/initial weight; ^4^Average daily gain; ^5^Average daily feed intake; ^6^Feed conversion ratio = feed intake/weight gain.

### Dietary effects of CN on serological indices in finishing pigs

Serum biochemical analyses of pigs fed the experimental diets are presented in Table 2. The results showed that pigs fed the CN supplemented diets had significantly higher HDLP levels than those fed the CON diet; however, other parameters such as GPT, GOT, BIL, TG, ALP, CHOL, and urea levels were unchanged at the end of 28 days of pigs. Furthermore, at the end of the 40 days, the serological data demonstrated that pigs fed the CN2 supplemented diets had significantly decreased levels of GPT, GOT, and CHOL compared to the CON diet. The TG level in pigs fed the CN1 and CN2 supplemented diets was significantly lower than the CON diet group. However, HDLP levels were significantly decreased in pigs fed CN supplemented diets, but BIL, ALP, and urea levels were unaltered in pigs fed experimental diets.

**Table 2:** Dietary curcumin nanospheres (CN) on serum biochemical parameters in finishing pigs for 40 days ^1^.

**Items**	**Dietary treatments**			***P-*value**
	**CON (no CN)**	**CN1 (1.0 ml/kg)**	**CN2 (2.0 ml/kg)**	
**Day 28**				
GPT^2^	21.8 ± 2.6^a^	19.1 ± 4.9^a^	18.9 ± 0.8^a^	0.5203
GOT^3^	16.1 ± 2.9^a^	14.1 ± 2.2^a^	11.6 ± 1.4^a^	0.1202
BIL^4^	0.5 ± 0.1^a^	0.5 ± 0.1^a^	0.6 ± 0.2^a^	0.0000
TG^5^	73.7 ± 3.3^a^	71.9 ± 3.2^a^	71.2 ± 2.1^a^	0.5858
ALP^6^	20 ± 1.0^a^	21 ± 1.0^a^	20 ± 1.0^a^	0.0000
HDLP^7^	32.8 ± 2.1^b^	40.9 ± 2.3^a^	40.0 ± 2.9^a^	0.0120
CHOL^8^	109.0 ± 2.0^a^	110.7 ± 2.5^a^	109.7 ± 2.9^a^	0.7251
Urea	27.0 ± 2.3^a^	27.4 ± 1.6^a^	27.9 ± 2.7^a^	0.8864
**Day 40**				
GPT	28.2 ± 2.7^a^	23.4 ± 0.7^a^	15.3 ± 2.1^b^	0.0006
GOT	19.7 ± 3.5^a^	16.4 ± 3.9^a, b^	10.6 ± 2.9^b^	0.0444
BIL	0.7 ± 0.1^a^	0.6 ± 0.1^a^	0.7 ± 0.4^a^	0.8966
TG	85.9 ± 1.3^a^	72.9 ± 3.4^b^	71.9 ± 2.5^b^	0.0009
ALP	20 ± 1.0^a^	21 ± 1.0^a^	20 ± 1.0^a^	0.0000
HDLP	36.1 ± 2.9^b^	42.7 ± 1.9^a^	46.1 ± 2.6^a^	0.0078
CHOL	108.7 ± 1.2^a^	111.7 ± 1.5^a^	102.7 ± 1.5^b^	0.0007
Urea	35.6 ± 2.0^a^	34.4 ± 1.8^a^	35.9 ± 1.5^a^	0.5908

^1^Values are mean ± SD from three replicate groups of pigs (*n* = 3), where mean ± SD in each row with different superscripts (a,b) are statistically significant at the 5% level of significance (*P* < 0.05).

^2^GPT, glutamic pyruvic transaminase (mg/dL); ^3^GOT, glutamic oxaloacetic transaminase (mg/dL); ^4^BIL, total bilirubin (mg/dL); ^5^TG, triglycerides (mg/dL); ^6^ALP, alkaline phosphatase (mg/dL); ^7^HDLP, high density lipoprotein (mg/dL); ^8^CHOL, cholesterol (mg/dL).

### Dietary effects of CN on nutritional composition and meat color in finishing pigs

The neck and longissimus dorsi muscle chemical compositions in terms of protein, lipid, and ash contents were not significantly affected by supplementation of CN in the pig diets compared to the CON diet (Tables 3, 4). However, moisture content of neck muscle was significantly lower and it was significantly higher in case of longissimus dorsi muscle in pigs fed the CN1 diet compared to the CON group of pigs. Meat characteristics of the neck muscle in terms of lightness, redness, and yellowness as well as lightness and yellowness in the longissimus muscle were not significantly affected by CN supplementation compared to the CON diet group; however, there was a tendency of increasing the redness in the longissimus dorsi muscle of pigs on higher CN supplementation (2 ml/kg) than that in the CON diet group (Tables 3, 4).

**Table 3:** Dietary curcumin nanospheres (CN) on meat quality of neck muscle in finishing pigs for 40 days^1^.

**Items**	**Dietary treatments**			***P-*value**
	**CON (no CN)**	**CN1 (1.0 ml/kg)**	**CN2 (2.0 ml/kg)**	
**Proximate composition**				
Moisture (%)	53.2 ± 0.2^a^	51.2 ± 0.1^b^	52.3 ± 0.9^ab^	0.0161
Protein (%)	14.8 ± 2.1^a^	15.2 ± 4.5^a^	15.4 ± 1.5^a^	0.9718
Lipid (%)	4.8 ± 0.3^a^	5.1 ± 0.2^a^	5.0 ± 0.2^a^	0.3877
Ash (%)	0.7 ± 0.1^a^	0.7 ± 0.2^a^	0.7 ± 0.1^a^	0.7461
**Meat color**				
L* (lightness)	41.1 ± 2.5^a^	46.4 ± 3.3^a^	41.3 ± 2.3^a^	0.0899
a* (redness)	16.9 ± 2.3^a^	19.4 ± 2.6^a^	19.7 ± 1.1^a^	0.2906
b* (yellowness)	11.4 ± 1.2^a^	11.5 ± 1.4^a^	12.1 ± 1.7^a^	0.0861

^1^Values are mean ± SD from three replicate groups of pigs (*n* = 3), where mean ± SD in each row with different superscripts (a,b) are statistically significant at the 5% level of significance (*P* < 0.05).

**Table 4:** Dietary curcumin nanospheres (CN) on meat quality of longissimus dorsi muscle in finishing pigs for 40 days^1^.

**Items**	**Dietary treatments**			***P-*value**
	**CON (no CN)**	**CN1 (1.0 ml/kg)**	**CN2 (2.0 ml/kg)**	
**Proximate composition**				
Moisture (%)	50.9 ± 0.6^b^	53.4 ± 1.1^a^	52.5 ± 1.2^ab^	0.0523
Protein (%)	18.2 ± 2.9^a^	15.6 ± 3.4^a^	15.0 ± 1.4^a^	0.3764
Lipid (%)	5.3 ± 0.2^a^	4.9 ± 0.2^a^	5.1 ± 0.3^a^	0.1810
Ash (%)	0.8 ± 0.1^a^	0.7 ± 0.1^a^	0.6 ± 0.1^a^	0.1842
**Meat color**				
L* (lightness)	49.9 ± 4.8^a^	52.7 ± 4.4^a^	50.1 ± 4.3^a^	0.7223
a* (redness)	6.2 ± 1.3^b^	8.4 ± 0.8^a, b^	11.5 ± 3.2^a^	0.0550
b* (yellowness)	6.4 ± 0.7^a^	8.4 ± 0.8^a^	8.3 ± 4.1^a^	0.0752

^1^Values are mean ± SD from three replicate groups of pigs (*n* = 3), where mean ± SD in each row with different superscripts (a,b) are statistically significant at the 5% level of significance (*P* < 0.05).

### Dietary effects of CN on meat carcass quality and grading of finishing pigs

The effects of CN on meat carcass quality and grading during finishing are presented in Table 5. The carcass weight and backfat thickness of pigs were significantly increased in pigs fed the high supplementation of CN diet; however, no significant differences in carcass weight and backfat thickness of pigs were observed between the low CN supplementation and CON diet groups. Furthermore, pigs fed the CN supplemented diets showed a gradual increase in the percentage of higher class meat grades (1+) as well as a gradual decline in the percentage of relatively lower class meat grades compared to the CON diets.

**Table 5:** Dietary curcumin nanospheres (CN) on carcass quality and grading in finishing pigs for 40 days^1^.

**Items**	**Dietary treatments**			***P-*value**
	**CON (no CN)**	**CN1 (1.0 ml/kg)**	**CN2 (2.0 ml/kg)**	
Carcass weight (kg)	81.00 ± 1.25^b^	81.03 ± 1.63^b^	82.23 ± 1.40^a^	0.001
Backfat thickness (mm)	20.80 ± 1.14^b^	20.80 ± 0.35^b^	21.33 ± 0.85^a^	0.001
**Grading**				
1+ (%)	23.33	33.33	36.67	
1 (%)	36.67	40.00	43.33	
2 (%)	40.00	26.67	20.00	

^1^Values are mean ± SD from three replicate groups of pigs (*n* = 3), where mean ± SD in each row with different superscripts (a,b) are statistically significant at the 5% level of significance (*P* < 0.05).

### Dietary effects of CN on fecal and intestinal bacterial contents in finishing pigs

The intestinal (jejunum) bacteria, *Lactobacillus* spp., *Escherichia coli* and *Salmonella* spp. in finishing pigs were not significantly affected by CN supplemented diets compared to the CON diet group (Table 6). The results showed that pigs fed the CN1 and CN2 supplemented diets had significantly lower fecal *Escherichia coli* contents than in those of pigs fed the CON diet. Moreover, pigs fed the CN2 diets had significantly lower fecal *E. coli* content than in the CN1 diet group. On the other hand, fecal *Lactobacillus* spp. and *Salmonella* spp. levels were unaltered in pigs fed the experimental diets (Table 6).

**Table 6:** Dietary curcumin nanospheres (CN) on intestinal and fecal bacteria counts in finishing pigs for 40 days^1^.

**Items**	**Dietary treatments**			***P-*value**
	**CON (no CN)**	**CN1 (1.0 ml/kg)**	**CN2 (2.0 ml/kg)**	
**Microbes in jejunum (CFU/g)**				
*Lactobacillus* spp.	9.78 ± 0.51^a^	9.89 ± 0.70^a^	9.67 ± 0.34^a^	0.880
*Escherichia coli*	3.67 ± 0.34^a^	3.89 ± 0.51^a^	3.89 ± 0.51^a^	0.801
*Salmonella* spp.	4.67± 0.58^a^	4.55± 0.69^a^	4.78± 0.19^a^	0.875
**Microbes in feces (CFU/g)**				
*Lactobacillus* spp.	6.89± 0.96^a^	6.11± 0.19^a^	6.33± 0.34^a^	0.332
*Escherichia coli*	7.45± 0.39^a^	5.55± 0.39^b^	4.44± 0.51^c^	0.001
*Salmonella* spp.	3.00± 0.33^a^	3.33± 0.34^a^	3.22± 0.19^a^	0.421

^1^Values are mean ± SD from three replicate groups of pigs (*n* = 3), where mean ± SD in each row with different superscripts (a,b,c) are statistically significant at the 5% level of significance (*P* < 0.05). CFU, colony forming unit.

### Dietary effects of CN on fecal noxious gas emissions in finishing pigs

Emission of fecal noxious gases such as ammonia was significantly reduced by dietary administration of CN in pigs compared to the CON diet; however, emission of fecal hydrogen sulfide gas was not significantly different among the pigs fed the experimental diets (Table 7).

**Table 7:** Dietary curcumin nanospheres (CN) on ammonia (NH_3_) and hydrogen sulfide (H_2_S) gas contents in the feces of finishing pigs for 40 days^1^.

**Items**	**Dietary treatments**			***P-*value**
	**CON (no CN)**	**CN1 (1.0 ml/kg)**	**CN2 (2.0 ml/kg)**	
Ammonia (ppm)	3.33 ± 1.04^a^	1.17 ± 0.76^b^	1.17 ± 0.29^b^	0.0200
Hydrogen sulfide (ppm)	0.17 ± 0.06^a^	0.13 ± 0.06^a^	0.17 ± 0.06^a^	0.7290

^1^Values are mean ± SD from three replicate groups of pigs (n=3), where mean ± SD in each row with different superscripts (a,b) are statistically significant at the 5% level of significance (*P* < 0.05).

### Dietary effects of CN on intestinal histomorphology of finishing pigs

**Figure 1:**
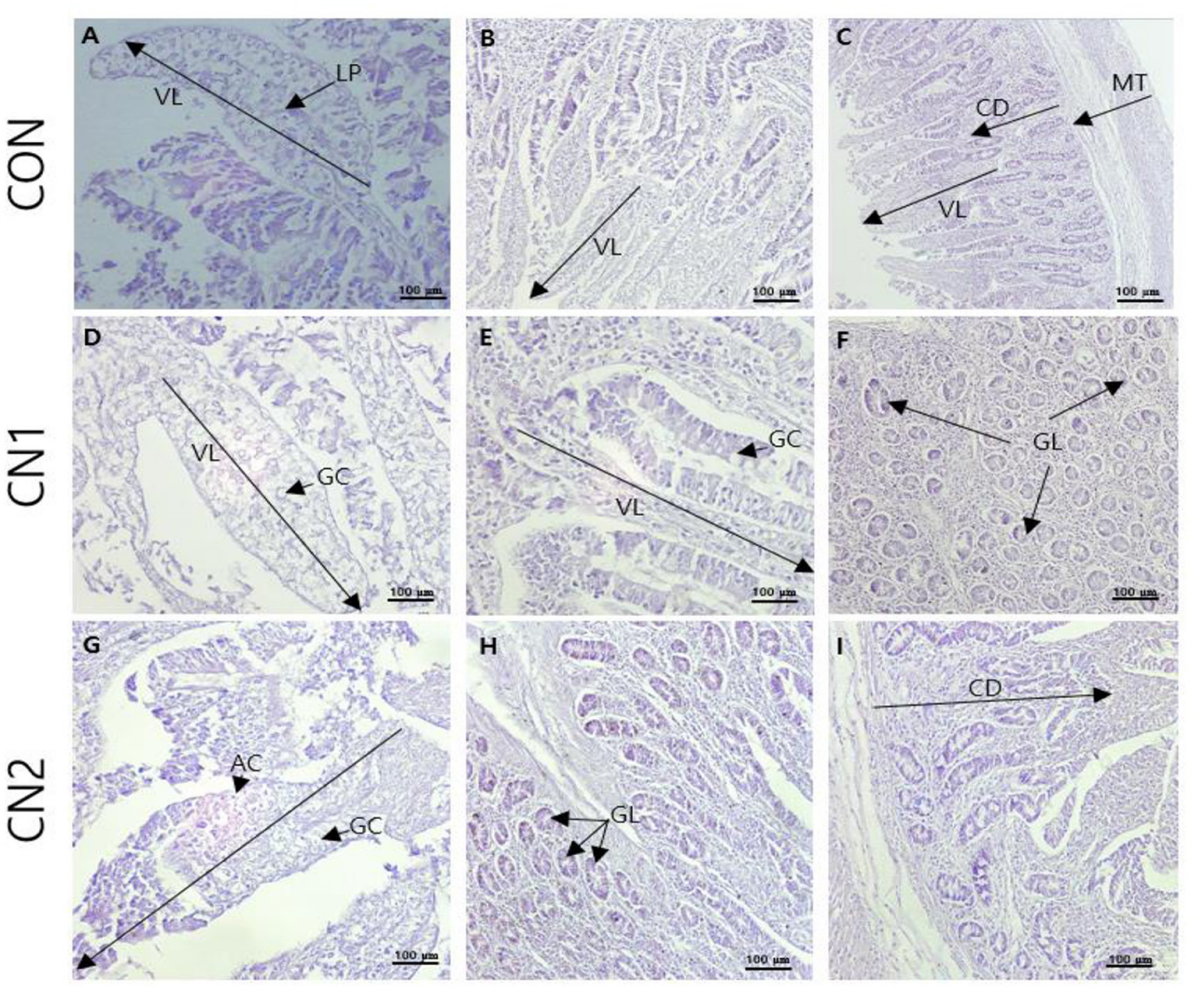
The representative photomicrographs from the jejunal sections of small intestine (*n* = 6) in finishing pigs fed **(A–C)** the control (CON) diet wihout curcumin nanospheres (CN); **(D–F)** the control diet supplemented with CN at 1.0 ml/kg diet (CN1); **(G–I)** the control diet supplemented with CN at 2.0 ml/kg diet (CN1); VL, villus length; LP, lamina propria; AC, absorptive cells; GC, goblet cells; CD, crypt depth; GL, mucosal glands; Hematoxylin and eosin **(H, E)** is used for tissue sections staining for viewing using bars 100 μm at different magnifications.

Histological sections from the jejunal intestine of finishing pigs fed the control and CN supplemented diets are shown in Figure 1. The results showed that villus height (VH) and crypt depth (CD) were significantly increased in pigs fed the CN supplemented diets compared to those fed the CON diet; however, there were no significant differences in the VH and CD of pigs fed the CN1 and CN2 diets (Table 8). Moreover, there were no significant differences in the VH/CD ratio or muscular thickness in pigs fed the experimental diets. The number of goblet cells was significantly higher in pigs fed the CN1 and CN2 diets than in the CON diet group.

**Table 8:** Dietary curcumin nanospheres (CN) on jejunal histomorphology in finishing pigs for 40 days^1^.

**Items**	**Dietary treatments**			***P-*value**
	**CON (no CN)**	**CN1 (1.0 ml/kg)**	**CN2 (2.0 ml/kg)**	
Villus height, VH (μm)	686.22 ± 3.67^b^	701.67 ± 2.65^a^	707.89 ± 4.67^a^	0.001
Crypt depth, CD (μm)	352.22 ± 3.56^b^	361.44 ± 1.17^a^	363.67 ± 2.91^a^	0.005
VH/CD	1.95 ± 0.03^a^	1.95 ± 0.03^a^	1.95 ± 0.03^a^	0.923
Muscular thickness (μm)	12.34 ± 1.15^a^	14.00 ± 1.00^a^	12.67 ± 2.34^a^	0.456
Goblet cells per villus	413.55 ± 1.35^c^	430.11 ± 1.65^b^	434.22 ± 1.17^a^	0.001

^1^Values are mean ± SD from three replicate groups of pigs (*n* = 3), where mean ± SD in each row with different superscripts (a,b,c) are statistically significant at the 5% level of significance (*P* < 0.05).

### Dietary effects of CN on intestinal immunohistochemistry of finishing pigs

**Figure 2:**
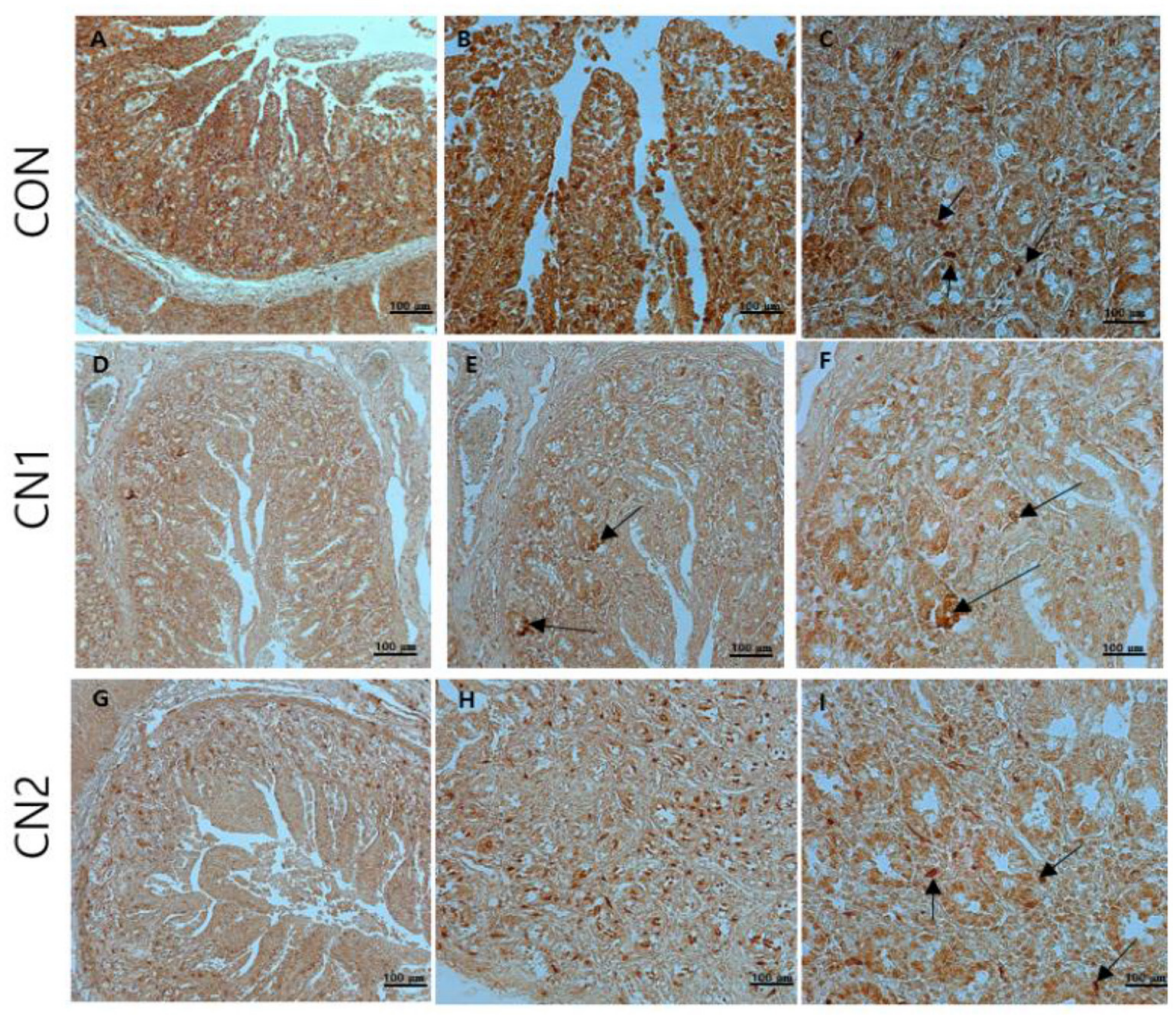
The immunohistochemical slides for the expressions of tumor necrosis factor-alpha (TNF-α) collected from the jejunal sections of small intestine (*n* = 6) in finishing pigs fed **(A–C)** the control (CON) diet wihout curcumin nanospheres (CN); **(D–F)** the control diet supplemented with CN at 1.0 ml/kg diet (CN1); **(G–I)** the control diet supplemented with CN at 2.0 ml/kg diet (CN1); The arrow heads represents the intensity of expressions of TNF-α cytokine. The 3,3'-diaminobenzidine (DAB) is used as the substrate for tissue sections staining for viewing using bars 100 μm at different magnifications.

**Figure 3:**
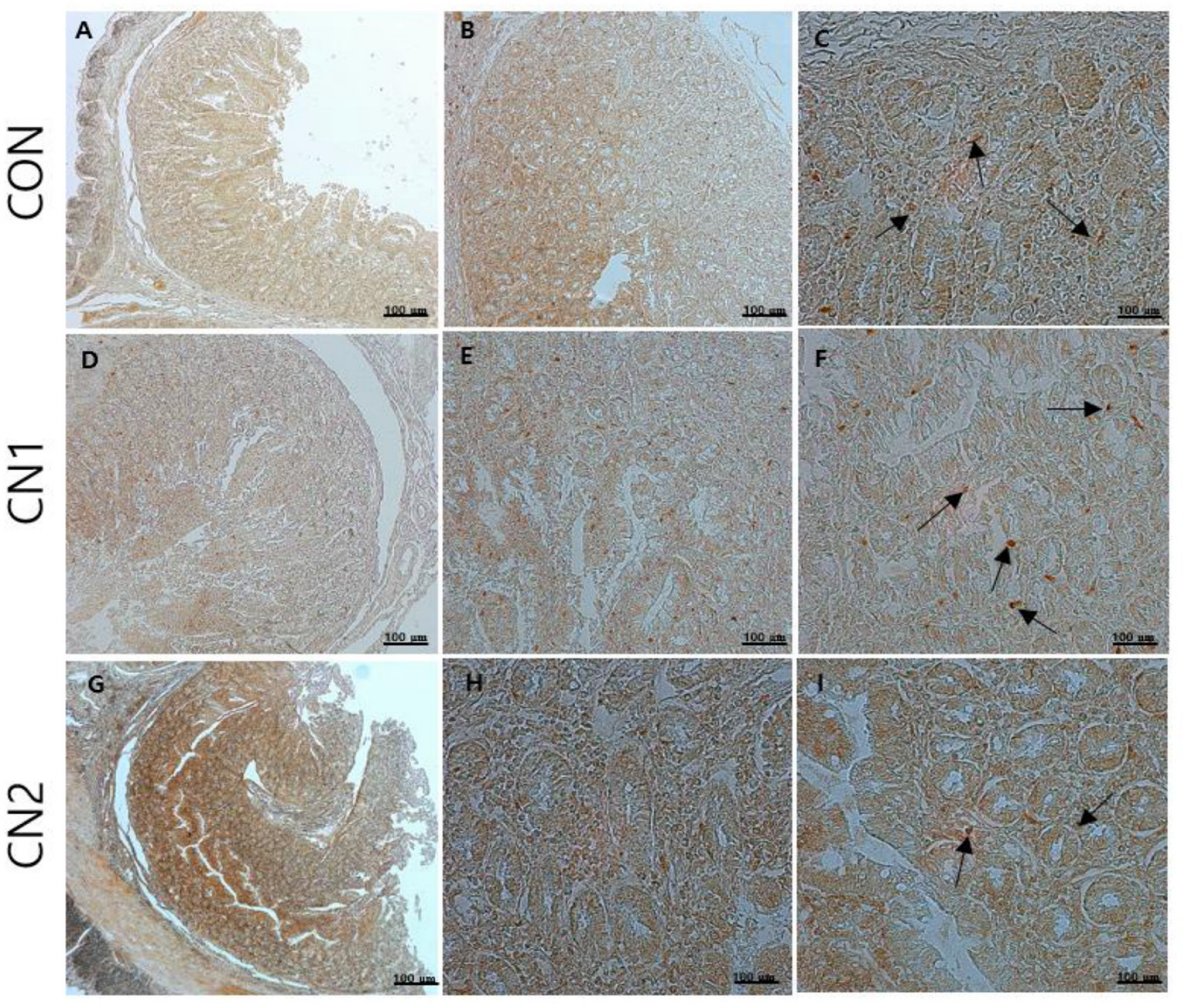
The immunohistochemical slides for the expressions of immunoglobulin A (IgA) collected from the jejunal sections of small intestine (*n* = 6) in finishing pigs fed **(A–C)** the control (CON) diet wihout curcumin nanospheres (CN); **(D–F)** the control diet supplemented with CN at 1.0 ml/kg diet (CN1); **(G–I)** the control diet supplemented with CN at 2.0 ml/kg diet (CN1); The arrow heads represents the intensity of expressions of IgA antibody. The 3,3'-diaminobenzidine (DAB) is used as the substrate for tissue sections staining for viewing using bars 100 μm at different magnifications.

**Figure 4:**
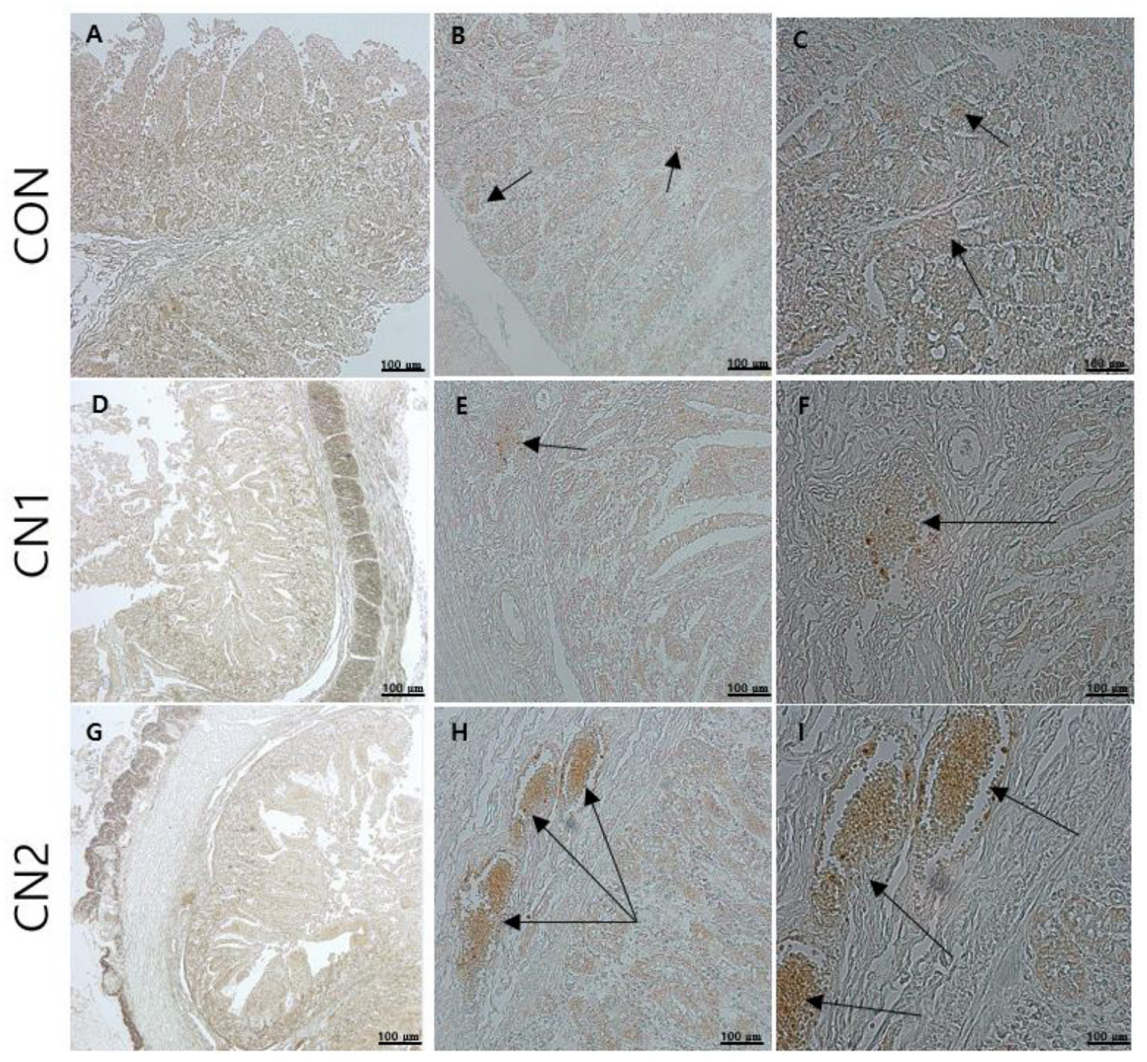
The immunohistochemical slides for the expressions of claudin 3 (CD3) collected from the jejunal sections of small intestine (*n* = 6) in growing to finishing pigs fed **(A–C)** the control (CON) diet wihout curcumin nanospheres (CN); **(D–F)** the control diet supplemented with CN at 1.0 ml/kg diet (CN1); **(G–I)** the control diet supplemented with CN at 2.0 ml/kg diet (CN1); The arrow heads represents the intensity of expressions of CD3 as the tight junction protein. Tissue sections were stained with 3,3'-diaminobenzidine (DAB) viewing using bars 100 μm at different magnifications.

Immunohistochemistry of jejunum sections of the intestine of finishing pigs demonstrated that the expression of TNF-α was reduced in the CN supplemented diet groups compared to the CON diet group (Figure 2). On the other hand, IgA and CD3 were found to be highly expressed in the intestine of pigs fed the CN1 and CN2 diets compared to the CON diet (Figures 3, 4) at different magnifications on the immunohistochemical slides.

## Discussion

Phytogenic compounds as natural feed additives have been extensively used as growth promoter and immune booster in the feedstuffs of monogastric farm animals such as pigs, poultry and fish (4). Recently, researchers have emphasized the use of phytogenic compounds in the form of nanocapsules because most phytogenic materials are poorly bioavailable in biological systems. For this, incorporation of phytogenic compounds in nano encapsulation form with liposomal nano carrier showed most effective drug delivery systems in murine as a monogastric animal model for biomedical applications (22–25). Furthermore, it has been reported that liposomal nano carrier with its spherical form can easily entrap the lipophilic and hydrophobic curcumin, and protect itself from external stress mediated by oxygen, pH and enzymatic degradation during digestion and absorption in gastrointestinal tract (26). Therefore, encapsulation of the phytogenic compounds in nano structure lead to the enahnced intraceullular uptake and effective delivery in the target organs through modifying the surface of nano carrier and increase the sensitivity of drug to the target areas (7). In line with previous studies on the utilization of nanotechnology in monogastric animals, the results of the current study showed the positive outcome of dietary curcumin nanospheres in terms of enhanced growth and feed utilization, improved serological indices, meat quality, and intestinal health status, as well as reduced pathogenic bacterial content and noxious gas in the feces of finishing pigs. Interestingly, the feeds administered with CN were well accepted by the pigs, and no mortality or moribund pigs were observed during the experimental period.

In this study, pigs fed CN supplemented diets showed remarkably higher growth in terms of FW, WG, and ADG at the end of the 40 day long rearing period. Interestingly, after 28 days, we found significantly higher growth performance in pigs fed diets with higher CN content (CN2). However, from 29 to 40 days, no significant differences were found in the growth of pigs fed CN supplemented or control diets. The results of this study indicate that early stage pigs are more capable of utilizing CN for growth than late stage pigs. In our previous study, we demonstrated the potential of low doses of CN in a weaned piglet model (12). In agreement with the present study, Marcon et al. (10) reported that dietary ethyl polymethacrylate nanocapsules loaded with curcumin (N-CU) at 1.89 mg/kg diet could facilitate weight gain in Lacaune lambs. However, researchers also found that higher inclusion of nanoencapsulated curcumin (4 mg/kg diet) had no effect on growth and health improvement in lambs (10). In addition, Ashry et al. (27) observed that a 20–30 mg/kg diet of curcumin could improve the health of Gilthead seabream fish as a monogastric animal model, which indicates that curcumin administered at more than 10 times would be equally effective as dietary nanocurcumin in animal feeds. This is in agreement with Shaikh et al. (3) who compared the efficacy of native curcumin and nanocurcumin in an animal model. Furthermore, Marchiori et al. (9) reported that dietary nanocurcumin administered at a dosage three times lower than that of free curcumin enhanced the growth performance and egg quality in quails. However, Rahmani et al. (28) revealed that curcumin and nanocurcumin had equal effects at the same concentrations in terms of growth enhancement and heat resistance in broiler chickens. In addition, Bao et al. (29) did not find any significant effect on the dietary supplementation of nanocurcumin on overall performance and feed utilization in juvenile largemouth bass fish. In agreement with the present study, Taghavinia et al. (25) found nanoliposome-loaded phenolics from *Nasturtium officinale* improved the average daily weight gain in mice. In relation to the curcumin nanobiotechnology in the present study, other researchers have proposed that nanoformulation of bioactive compounds such as microencapsulated organic acids, zinc oxide nanoparticles and coated sodium butyrate can be beneficial for enhancing growth and immunity in pigs (30–32). Regarding feed utilization, the results of the present study showed no significant effects of dietary CN on ADFI and FCR in finishing pigs which is in agreement with Marcon et al. (10). In contrast, in our previous study, we found positive effects of dietary CN on feed efficiency and FCR in weaned piglets, which was attributed to the fact that young pigs were more efficient in feed utilization than finishing pigs.

Serological information of blood is an important tool for ascertaining the health status of animals. In this study, at the end of 28 days, serological parameters, such as GPT, GOT, BIL, TG, ALP, CHOL, and urea were unaffected by dietary CN1 or CN2 supplementation in finishing pigs. However, GPT, GOT, TG and CHOL levels decreased significantly at the end of the finishing stage in pigs fed CN incorporated diets, which might be attributed to potential effects of dietary CN in terms of improving health condition of pigs. In accordance with the present study, Marcon et al. (10) corroborated that dietary curcumin nanocapsules can decrease the blood TG levels in lambs. Moreover, Reda et al. (15) postulated that dietary nanocurcumin can increase serum HDLP levels and decrease TG, CHOL, GPT (or ALT, alanine aminotransferase), and GOT (or AST, aspartate aminotransferase), and did not affect urea levels in Japanese quails which supports the data of the current study. Likewise, it is reported that phytogenic phenolic compounds loaded with nanoliposome can reduce blood ALT, AST and ALP levels on induced cadmium toxicity in mice (24).

In this study, we evaluated how dietary CN supplementation impact on the quality of meat in the neck and longissimus dorsi muscle of finishing pigs. The results showed that proximate compositions in terms of protein, lipid, and ash content of neck and longissimus dorsi muscles were not significantly affected by dietary CN. In addition, the meat color of the neck and longissimus dorsi muscles did not significantly change based on the lightness and yellowness of the meat samples. Interestingly, the redness of the longissimus dorsi muscle was highly increased upon increasing the dose of dietary CN in finishing pigs, which is in agreement with the data reported on the effects of dietary curcumin on the longissimus dorsi muscle of finishing pigs, as well as the breast muscles of broiler and duck meats (33–35). The carcass weight and backfat thickness of pigs fed the higher CN supplemented (2.0 ml/kg diet) diet were found to be higher than those of the control and low concentration CN groups, which endorsed the beneficial effects of CN supplementation on weight gain after slaughtering the pigs. Similarly, Reda et al. (15) observed an improvement in carcass traits upon supplementation with dietary curcumin nanoparticles in Japanese quails. In addition, in the present study, the grading percentage (1+) of pork meat also increased with dietary supplementation of CN, which was attributed to the upgradation of meat quality in pigs fed CN incorporated diets. The findings of the current study regarding the meat quality of pork are consistent with those of Zhang et al. (33) and Jin et al. (35) for broiler and duck meat, respectively, which were supplemented with dietary curcumin.

In this study, we reported the effects of dietary supplementation with CN on the pathogenic and beneficial bacterial contents in fecal and intestinal samples collected from finishing pigs at the end of the experimental period. The results revealed that dietary CN had no effects on pathogenic bacteria, *Escherichia coli* and *Salmonella* spp., or the beneficial bacterium, *Lactobacillus* spp. in the jejunal samples after slaughtering the pigs. However, we observed that pathogenic *E. coli* bacterial content in feces was drastically reduced in pigs fed CN supplemented diets compared to those fed the control diet. These results are in accordance with those of Muniyappan et al. (31), who postulated that dietary microencapsulated organic acids can reduce the *E. coli* bacterial content in the feces of growing to finishing pigs. Furthermore, Reda et al. (15) confirmed that dietary nanocurcumin significantly depleted *E. coli* and *Salmonella* spp. and augmented lactic acid bacterial counts in the caeca of growing Japanese quails. In addition, Sampath et al. (36) reported that dietary black piper plant extract can linearly increase *Lactobacillus* and decrease *E. coli* bacterial counts in the fecal samples of finishing pigs. Lesschen et al. (37) reported that NH_3_ and H_2_S are important noxious air pollutants emitted from livestock farms. In the present study, we found that fecal gas content, such as ammonia, was significantly reduced in finishing pigs fed CN compared to the control diet. Likewise, Moniruzzaman et al. (12) found that dietary CN reduced fecal ammonia gas content in weaned piglets. In accordance with the present study, it has been reported that dietary plant extracts can reduce ammonia gas emissions from the feces of finishing pigs (38, 39). In contrast, Muniyappan et al. (31) observed an insignificant effect of microencapsulated organic acids on the ammonia gas content in the feces of growing to finishing pigs.

Gut morphology can serve as an important tool to evaluate the absorption and utilization of a feed additive in the intestine, which ultimately affect the growth and health status of animals. Abnormalities or changes in gastrointestinal tract (GIT) especially in small intestine as the major site for nutrient absorption may influence the overall growth of the animals. In the current study, pigs fed the CN supplemented diets showed remarkably enhanced villus length, crypt depth, and goblet cell number in the jejunal part of intestine of finishing pigs, which was attributed to the higher surface area of the intestine for absorption of curcumin. These results are in accordance with those of Upadhaya et al. (32) and Lei and Kim (40), who reported that dietary coated sodium butyrate and coated zinc oxide could increase villus length and crypt depth in pigs, respectively. Likewise, Xun et al. (41) observed that dietary curcumin could enhance villus height, crypt depth, and goblet cell numbers in weaned piglets. In agreement of our study, Taghavinia et al. (21) and Beyrami et al. (24) found that plant derived phenolic compounds with liposomal nano carrier can improve the intestinal health on induced colorectal cancer or metal toxicity in murine model, respectively.

Immunohistochemistry (IHC) is the most frequently used method for immunostaining selective antigen proteins by binding with specific antibody proteins in animal tissues (42). Chromogenic IHC is a widely used method in which an antibody is conjugated with a peroxidase enzyme that executes a color producing reaction (42). The GIT of animals is composed of the outermost cellular barrier and the innermost immune functional barrier systems. For the intestinal epithelial cell barrier functions, tight junction proteins such as claudins, occludin and zona occludin-1 are key proteins to create a physiological and immunological barrier in the intestine. However, disruption or reduction of tight junction protein concentrations may cause a leaky gut, which may ultimately affect intestinal permeability in terms of digestion and absorption of nutrients (43). Gut associated lymphoid tissue (GALT) has the potential to mediate innate and adaptive mucosal immune responses. Tumor necrosis factor-α (TNFα) has an important intermediary role in GALT growth and antimicrobial defense mechanisms, and it functions as a pro-inflammatory regulator (44). Immunoglobulin A (IgA) is a major antibody class that plays a principal role in capturing pathogenic microorganisms and helps to maintain intestinal homeostasis as the primary defense system (41). In the present study, the chromogenic expression of TNFα was reduced in the jejunal intestine of pigs fed CN supplemented diets in relation to the control diet. On the other hand, the expression of IgA and CD3 proteins was increased in the jejunal intestine of pigs fed CN diets, which was attributed to the enhancement of gut immunity and intestinal permeability of curcumin in finishing pigs. In line with the present study, Xun et al. (41) found that dietary supplementation of curcumin at 300 mg/kg or 400 mg/kg diet could improve the intestinal barrier integrity and immune status in terms of increasing the expression of sIgA (secretory IgA) and interleukin 10 (IL-10) as well as decreasing the mRNA expression of TNFα and Toll-like receptor 4 (TLR4) in jejunal mucosa of weaned piglets. Likewise, it has been reported that quercetin at 25 mg/kg diet can improve intestinal oxidative status and inflammation by increasing villi height and enhancing the mRNA expression of occludin and zonula occudens-1 (ZO-1) as well as reducing the intestinal reactive oxygen species in the jejunum of finishing pigs on transport stress (45). Shi et al. (46) found that dietary single supplementation of curcumin at higher dose (200 mg/kg) or lower dose of curcumin (200 mg/kg) in combination with piperine (25 mg/kg) can improve the intestinal permeability in terms of increasing the mRNA expression of occludin, claudin-1 and zonula occludin-1 in the jejunal and ileal mucosa of weaned Wuzhishan piglets. In addition, Reda et al. (15) corroborated that dietary nanocurcumin can enhance immunoglobulin G (IgG) and immunoglobulin M (IgM) levels in growing Japanese quails, which supported the immunostaining features of IgA protein expression in the jejunum of finishing pigs fed CN supplemented diets in the present study.

## Conclusion

In conclusion, the results of the present study showed that dietary supplementation with curcumin nanospheres can enhance the growth, serological indices, immunity, meat quality, gastrointestinal morphology, and reduce intestinal and fecal infectious bacterial colonies, as well as fecal noxious gas (ammonia) content in finishing pigs.

## Data availability statement

The original contributions presented in the study are included in the article/supplementary material, further inquiries can be directed to the corresponding author.

## Ethics statement

The study was approved by the Animal Care and Use Committee of Jeju National University, Jeju Island, Republic of Korea (approval no. 2021-0056).

## Author contributions

MM has planned the experiment, determined the growth, somatic indices, hematology, meat quality, immunohistochemistry, microbiological analyzes, fecal gas analyzes, drafted the final article, and analyzed the statistical data and finalized the manuscript. DK did sample analyzes, immunohistochemistry, and drafting of the manuscript. HK, NK, SC, and AK growth, somatic indices, hematology, meat quality, and fecal gas analyses. KH and TM critically supervised and helped in experimental planning with the addition of manuscript drafting and finalizing. All authors read and approved the final manuscript.

## References

[1] Johannah NM Ashil J Balu M Krishnakumar IM. Dietary addition of a standardized extract of turmeric (TurmaFEED TM) improves growth performance and carcass quality of broilers. J Anim Sci Technol. (2018) 60:1–9. 10.1186/s40781-018-0167-7 29854411 PMC5971416

[2] Yazdi FG Soleimanian-Zad S Van den Worm E Folkerts G. Turmeric extract: potential use as a prebiotic and anti-inflammatory compound? Plant Food Hum Nutr. (2019) 74:293–9. 10.1007/s11130-019-00733-x 31098880

[3] Shaikh J Ankola DD Beniwal V Singh D Kumar MNVR. Nanoparticle encapsulation improves oral bioavailability of curcumin by at least 9-fold when compared to curcumin administered with piperine as absorption enhancer. Eur J Pharm Sci. (2009) 37:223–30. 10.1016/j.ejps.2009.02.019 19491009

[4] Moniruzzaman M Min TS. Curcumin, curcumin nanoparticles and curcumin nanospheres: a review on their pharmacodynamics based on monogastric farm animal, poultry and fish nutrition. Pharmaceutics. (2020) 12:447. 10.3390/pharmaceutics12050447 32403458 PMC7284824

[5] Karthikeyan A Kim NY Moniruzzaman M Beyene AM Do KT Senthil KS. Curcumin and its modified formulations on inflammatory bowel disease (IBD); the story so far and future outlook. Pharmaceutics. (2021) 13:484. 10.3390/pharmaceutics13040484 33918207 PMC8065662

[6] Kharat M McClements DJ. Recent advances in colloidal delivery systems for nutraceuticals: a case study—delivery by design of curcumin. J Colloid Int Sci. (2019) 557:506–18. 10.1016/j.jcis.2019.09.045 31542691

[7] Tabatabaeain SF Karimi E Hashemi M. *Satureja khuzistanica* essential oil-loaded solid lipid nanoparticles modified with chitosan-folate: evaluation of encapsulation efficiency, cytotoxic and pro-apoptotic properties. Front Chem. (2022) 10:904973. 10.3389/fchem.2022.904973 35815210 PMC9257980

[8] Jaguezeski AM Gündel SS Favarin FR Gündel A Souza CF Baldissera MD. Low-dose curcumin-loaded Eudragit L-100-nanocapsules in the diet of dairy sheep increases antioxidant levels and reduces lipid peroxidation in milk. J Food Eng. (2019) 43:e12942. 10.1111/jfbc.12942 31368562

[9] Marchiori MS Oliveira RC Souza CF Baldissera MD Ribeiro QM Wagner R. Curcumin in the diet of quail in cold stress improves performance and egg quality. Anim Feed Sc. Technol. (2019) 254:144–57. 10.1016/j.anifeedsci.2019.05.015

[10] Marcon H Griss LG Molosse VL Cecere BGO Alba DF Leal KW. S. Dietary supplementation with curcumin-loaded nanocapsules in lambs: nanotechnology as a new tool for nutrition. Anim Nutr. (2021) 7:521–9. 10.1016/j.aninu.2020.06.014 34258441 PMC8245810

[11] Alagawany M Farag MR Abdelnour SA Dawood MAO Elnesr SS Dhama K. Curcumin and its different forms: a review on fish nutrition. Aquaculture. (2021) 532:736030. 10.1016/j.aquaculture.2020.736030

[12] Moniruzzaman M Kim HH Shin HW Kim HS Kim NY Chin SY. Evaluation of dietary curcumin nanospheres in a weaned piglet model. Antibiotics. (2021) 10:1280. 10.3390/antibiotics10111280 34827218 PMC8614963

[13] Abdel-Tawwab M Eissa ESH Tawfk WA Elnabi HEA Saadony S Bazina WK Ahmed RA. Dietary curcumin nanoparticles promoted the performance, antioxidant activity, and humoral immunity, and modulated the hepatic and intestinal histology of Nile tilapia fingerlings. Fish Physiol. Biochem. (2022) 48:585–601. 10.1007/s10695-022-01066-4 35380335 PMC9156469

[14] Oroumieh SK Vanhaecke L Valizadeh R Meulebroek LV Naserian AA. Effect of nanocurcumin and fish oil as natural anti-inflammatory compounds vs. glucocorticoids in a lipopolysaccharide inflammation model on Holstein calves' health status. Heliyon. (2021) 7:e05894. 10.1016/j.heliyon.2020.e05894 33553719 PMC7855347

[15] Reda FM El-Saadiny MT Elnesr SS Alagawany M Tufarelli V. Effect of dietary supplementation of biological curcumin nanoparticles on growth and carcass traits, antioxidant status, immunity and caecal microbiota of Japanese quails. Animals. (2020) 10:754. 10.3390/ani10050754 32357410 PMC7277682

[16] Kim JY Lee YM Kim DW Min TS Lee SJ. Nanosphere loaded with curcumin inhibits the gastrointestinal cell death signaling pathway induced by the foodborne pathogen *Vibrio vulnificus*. Cells. (2020) 9:631. 10.3390/cells9030631 32151068 PMC7140471

[17] Kim JY Min TS Lee SJ. Nanospheres loaded with curcumin promote gut epithelial motility through F-actin-related migration signaling events. J Nutr Biochem. (2021) 88:108555. 10.1016/j.jnutbio.2020.108555 33249186

[18] Kim JI Sohn YG Jung JH Park YI. Genetic parameter estimates for backfat thickness at three different sites and growth rate in swine. Asian-Australas J Anim Sci. (2004) 17:305–8. 10.5713/ajas.2004.305

[19] KAPE-Korea Institute for Animal Products Quality Evaluation. Animal Products Grade System: The Pork Carcass Grading System. (2010). Available online at: https://www.ekape.or.kr/index.do (accessed on November 10, 2021).

[20] Association of Official Analytical Chemists (AOAC). Official Methods of Analysis, 16th ed. Arlington, VA: Association of Official Analytical Chemists (AOAC) (1995).

[21] Tian Z Cui Y Lu H Ma X. Effects of long-term feeding diets supplemented with *Lactobacillus reuteri* 1 on growth performance, digestive and absorptive function of the small intestine in pigs. J Func Foods. (2020) 71:104010. 10.1016/j.jff.2020.104010

[22] Moeini S Karimi E Oskoueian E. Antiproliferation effects of nanophytosome-loaded phenolic compounds from fruit of *Juniperus polycarpos* against breast cancer in mice model: synthesis, characterization and therapeutic effects. Cancer Nanotech. (2022) 13:20. 10.1186/s12645-022-00126-x

[23] Poorbagher MRM Karimi E Oskoueian E. Hepatoprotective effect of nanoniosome loaded *Myristica fragrans* phenolic compounds in mice-induced hepatotoxicity. J Cell Molec Med. (2022) 2022:5517–27. 10.1111/jcmm.17581 36226354 PMC9639044

[24] Beyrami M Karimi E Oskoueian E. Synthesized chrysin-loaded nanoliposomes improves cadmium-induced toxicity in mice. Environ Sci Pollut Res. (2020) 27:40643–51. 10.1007/s11356-020-10113-7 32671712

[25] Taghavinia F Teymouri F Farokhrouz F Bagherabad EH Farjami S Karimi E. Nanoliposome-loaded phenolics from *Nasturtium officinale* improves health parameters in a colorectal cancer mouse model. Animals. (2022) 12:3492. 10.3390/ani12243492 36552412 PMC9774266

[26] Hasan M Belhaj N Benachour H Barberi Heyob M Kahn CJF Jabbari E. Liposome encapsulation of curcumin: physico-chemical characterizations and effects on MCF7 cancer cell proliferation. Int J Pharm. (2014) 461:519–28. 10.1016/j.ijpharm.2013.12.007 24355620

[27] Ashry AM Hassan AM Habiba MM El-Zayat A El-Sharnouby ME Sewilam H. The impact of dietary curcumin on the growth performance, intestinal antibacterial capacity, and haemato-biochemical parameters of gilthead seabream (*Sparus aurata*). Animals. (2021) 11:1779. 10.3390/ani11061779 34203579 PMC8232219

[28] Rahmani M Golian A Kermanshahi H Bassami MR. Effects of curcumin and nanocurcumin on growth performance, blood gas indices and ascites mortalities of broiler chickens reared under normal and cold stress conditions. Italian J Anim Sci. (2017) 16:438–46. 10.1080/1828051X.2017.1290510

[29] Bao X Chen M Yue Y Liu H Yang Y Yu H. Effects of dietary nano-curcumin supplementation on growth performance, glucose metabolism, and endoplasmic reticulum stress in juvenile largemouth bass, *Micropterus salmoides*. Front Mar Sci. (2022) 9:924569. 10.3389/fmars.2022.924569

[30] Milani NC Sbardella M Ikeda NY Arno A Mascarenhas BC Miyada VS. Dietary zinc oxide nanoparticles as growth promoter for weanling pigs. Anim Feed Sci Technol. (2017) 227:13–23. 10.1016/j.anifeedsci.2017.03.001

[31] Muniyappan M Palanisamy T Kim IH. Effect of microencapsulated organic acids on growth performance, nutrient digestibility, blood profile, fecal gas emission, fecal microbial, and meat-carcass grade quality of growing-finishing pigs. Livest Sci. (2021) 252:104658. 10.1016/j.livsci.2021.104658

[32] Upadhaya SD Jiao Y Kim YM Lee KY Kim IH. Coated sodium butyrate supplementation to a reduced nutrient diet enhanced the performance and positively impacted villus height and faecal and digesta bacterial composition in weaner pigs. Anim Feed Sci Technol. (2020) 265:114534. 10.1016/j.anifeedsci.2020.114534

[33] Zhang J Hu Z Lu C Bai K Zhang L Wang T. Effect of various levels of dietary curcumin on meat quality and antioxidant profile of breast muscle in broilers. J Agric Food Chem. (2015) 63:3880–6. 10.1021/jf505889b 25823972

[34] Zhang J Yan E Zhang L Wang T Wang C. Curcumin reduces oxidative stress and fat deposition in longissimus dorsi muscle of intrauterine growth-retarded finishing pigs. Anim Sci J. (2022) 93:e13741. 10.1111/asj.13741 35707899

[35] Jin S Yang H Liu F Pang Q Shan A Feng X. Effect of dietary curcumin supplementation on duck growth performance, antioxidant capacity and breast meat quality. Foods. (2021) 10:2981. 10.3390/foods10122981 34945532 PMC8701154

[36] Sampath V Shanmugam S Park JH Kim IH. The effect of black pepper (*Piperine*) extract supplementation on growth performance, nutrient digestibility, fecal microbial, fecal gas emission, and meat quality of finishing pigs. Animals. (2020) 10:1965. 10.3390/ani10111965 33113842 PMC7693949

[37] Lesschen JP van den Berg M Westhoek HJ Witzke HP Oenema O. Greenhouse gas emission profiles of European livestock sectors. Anim Feed Sci Technol. (2011) 166:16–28. 10.1016/j.anifeedsci.2011.04.058

[38] Dang DX Kim YM Kim IH. Effects of a root extract from *Achyranthes Japonica Nakai* on the growth performance, blood profile, fecal microbial community, fecal gas emission, and meat quality of finishing pigs. Livest Sci. (2020) 239:104160. 10.1016/j.livsci.2020.104160

[39] Dang DX Kim IH. Effects of dietary supplementation of *Quillaja saponin* on growth performance, nutrient digestibility, fecal gas emissions, and meat quality in finishing pigs. J Appl Anim Res. (2020) 48:397–401. 10.1080/09712119.2020.1813739

[40] Lei XJ Kim IH. Evaluation of coated zinc oxide in young pigs challenged with enterotoxigenic *Escherichia coli* K88. Anim Feed Sci Technol. (2020) 262:114399. 10.1016/j.anifeedsci.2020.114399

[41] Xun W Shi L Zhou H Hou G Cao T Zhao C. Effects of curcumin on growth performance, jejunal mucosal membrane integrity, morphology and immune status in weaned piglets challenged with enterotoxigenic *Escherichia coli*. Int Immunopharm. (2015) 27:46–52. 10.1016/j.intimp.2015.04.038 25937483

[42] Ramos-Vara JA Miller MA. When tissue antigens and antibodies get along: revisiting the technical aspects of immunohistochemistry–the red, brown, and blue technique. Vet Pathol. (2014) 51:42–87. 10.1177/0300985813505879 24129895

[43] Wijtten PJA van der Meulen J Verstegen MWA. Intestinal barrier function and absorption in pigs after weaning: a review. Br J Nutri. (2011) 105:967–1. 10.1017/S0007114510005660 21303573

[44] Amevor FK Cui Z Du X Ning Z Deng X Xu D. Supplementation of dietary quercetin and vitamin e promotes the intestinal structure and immune barrier integrity in aged breeder hens. Front Immunol. (2022) 13:860889. 10.3389/fimmu.2022.860889 35386687 PMC8977514

[45] Zou Y Wei HK Xiang QH Wang J Zhou YF Peng J. Protective effect of quercetin on pig intestinal integrity after transport stress is associated with regulation oxidative status and inflammation. J Vet Med Sci. (2016) 78:1487–94. 10.1292/jvms.16-0090 27301842 PMC5059377

[46] Shi L Xun W Peng W Hu H Cao T Hou G. Effects of the single and combined use of curcumin and piperine on growth performance, intestinal barrier function and antioxidant capacity of weaned Wuzhisan piglets. Front Vet Sci. (2020) 7:418. 10.3389/fvets.2020.00418 32851010 PMC7411177

